# Potential yields and emission reductions of biojet fuels produced via hydrotreatment of biocrudes produced through direct thermochemical liquefaction

**DOI:** 10.1186/s13068-019-1625-2

**Published:** 2019-12-05

**Authors:** Susan van Dyk, Jianping Su, Mahmood Ebadian, Don O’Connor, Michael Lakeman, Jack (John) Saddler

**Affiliations:** 1 Forest Products Biotechnology/Bioenergy Group, The University of British Columbia, Vancouver, BC V6T 1Z4 Canada; 2 IEA Bioenergy, Task 39, Paris, France; 3 (S&T) Squared Consultants Inc., Delta, BC V4E 2Z2 Canada; 4 Boeing, Seattle, USA

**Keywords:** Biocrudes, Hydrotreatment, Pyrolysis, Hydrothermal liquefaction, Biojet fuel

## Abstract

**Background:**

The hydrotreatment of oleochemical/lipid feedstocks is currently the only technology that provides significant volumes (millions of litres per year) of “conventional” biojet/sustainable aviation fuels (SAF). However, if biojet fuels are to be produced in sustainably sourced volumes (billions of litres per year) at a price comparable with fossil jet fuel, biomass-derived “advanced” biojet fuels will be needed. Three direct thermochemical liquefaction technologies, fast pyrolysis, catalytic fast pyrolysis and hydrothermal liquefaction were assessed for their potential to produce “biocrudes” which were subsequently upgraded to drop-in biofuels by either dedicated hydrotreatment or co-processed hydrotreatment.

**Results:**

A significant biojet fraction (between 20.8 and 36.6% of total upgraded fuel volume) was produced by all of the processes. When the fractions were assessed against general ASTM D7566 specifications they showed significant compliance, despite a lack of optimization in any of the process steps. When the life cycle analysis GHGenius model was used to assess the carbon intensity of the various products, significant emission reductions (up to 74%) could be achieved.

**Conclusions:**

It was apparent that the production of biojet fuels based on direct thermochemical liquefaction of biocrudes, followed by hydrotreating, has considerable potential.

## Background

Currently, air transport accounts for approximately 2% of global carbon dioxide (CO_2_) emissions and these emissions are expected to continue to increase based on the sector’s steady growth [1]. Although aviation-related emissions can be partially reduced by strategies such as more efficient aircraft operations, infrastructure improvements, modernized air traffic management systems, etc., for the foreseeable future, aviation will be uniquely dependent on the use of low carbon-intensity biojet/sustainable aviation fuels to achieve significant reductions in aviation emissions by 2050 [2]. The emissions reduction potential of different biojet fuels can vary significantly, based on different feedstocks and technology pathways, with reported values ranging from 50 to 95% reduction potential as compared to conventional jet fuel [3–7]. As well as differences in the feedstock and technologies, the specific location where the drop-in biofuels are produced can also influence the carbon intensity of the biojet fraction as local utilities and infrastructure may differ significantly, e.g. electricity derived from coal, hydro, nuclear or natural gas.

The vast majority of biojet fuels that have been used to date are produced via the “conventional” hydrotreatment of oleochemicals/lipid feedstocks, including fats, oils and greases (FOGs) [8, 9]. However, these oleochemical feedstocks are usually expensive, limited in their availability and some of them come with sustainability concerns [10, 11]. Thus, to provide the volumes that will be required to meet the aspirational goals set by groups such as the Air Transport Action Group [1], processes based on more-abundant and cheaper biomass feedstocks will be required. Previous work that looked at the potential to produce drop-in biofuels from biomass suggested that thermochemical processes [11], such as gasification and liquefaction, are the technologies most likely to be used to produce drop-in biofuels such as biojet fuel. As summarized earlier [11], although gasification of biomass and subsequent Fischer–Tropsch synthesis to liquid biofuels is technically feasible, the large scale that will likely have to be adopted to make this approach economically attractive will make biomass supply chains more challenging while high capital costs are also likely to be encountered [11]. As work on the gasification route continues, some of the liquefaction technologies such as pyrolysis and hydrothermal liquefaction have reached the pilot, demonstration or small-commercial stage of development. These processes are now in a position to provide enough of the “biocrude” feedstock to allow an assessment of the upgrading processes that could be used to produce finished fuels such as biojet fuel and allow an assessment of the carbon reduction potential of producing and using this lower carbon intensity biojet fuel.

In the work reported here, biocrudes from three direct thermochemical liquefaction processes [fast pyrolysis (FP), catalytic fast pyrolysis (CFP), and hydrothermal liquefaction (HTL)], were sourced (two, 50-l volumes of each biocrude) and subsequently upgraded by hydrotreating using two different hydroprocessing approaches, “dedicated” or “co-processed” hydrotreatment. The Pacific Northwest National Laboratories (PNNL) used a “dedicated” hydrotreating approach using pure biocrude while Canmet-Energy Ottawa (Canmet) used a co-processed hydrotreating approach, as described in “Methods”. As very limited prior work has been published on the feasibility of producing biojet fuels via these pathways we wanted to determine whether this general approach had potential to be a viable route to producing biojet fuels while also assessing the emission reduction potential of each process [4, 6].

The most established biocrudes that are produced via fast pyrolysis were anticipated to encounter some upgrading challenges, primarily due to their relatively high oxygen content, total acid number (TAN) levels and relative instability [12, 13] while alternative thermochemical liquefaction technologies, such as catalytic pyrolysis, hydropyrolysis and hydrothermal liquefaction have been reported to produce biocrudes with improved characteristics such as a lower oxygen content [14–17]. Consequently, it has been suggested that these biocrudes might be more readily upgraded into finished biofuels due to their greater stability and lower hydrogen requirements [17, 18]. To date, there have been very few studies that have compared the different direct thermochemical liquefaction processes used to make biocrudes and how these biocrudes might be upgraded to finished fuels [19]. As well as assessing the technical feasibility of producing biojet fuels from biocrudes, we also carried out an initial assessment of the carbon intensity of the resulting fuels. When the GHGenius life cycle analysis model was used to assess the carbon intensity of the various products, significant emission reductions (up to 74%) could be achieved. As described in more detail below, this study demonstrated that direct thermochemical liquefaction biocrudes could be used to produce lower carbon intensity biojet fuels via a hydrotreatment upgrading approach.

## Results and discussion

### Biocrude production and characterization

As mentioned earlier, fast pyrolysis is a well-established process that is used for the direct thermochemical liquefaction of biomass to biocrudes. This approach has been commercialized by companies such as Ensyn and BTG (Biomass Technology Group BV) [8, 12]. As one of the objectives of the work was to obtain enough material (nominally 50 l) that could be used for subsequent upgrading trials, a fast pyrolysis biocrude was obtained from BTG where softwood had been used as the feedstock. A softwood-derived biocrude produced by catalytic pyrolysis was also obtained from VTT while a hydrothermal liquefaction derived biocrude, also made from softwoods, was obtained from Aarhus University. BTG operates at a small commercial scale while the other facilities are both at the pilot scale. When the composition of the three biocrudes was assessed (Table 1) the fast pyrolysis biocrude was shown to be typical of previously published data [20, 21], showing a high oxygen (47.5 wt% wet basis, 35.2 wt% dry basis) and water content (23.5 wt%), a low net heat of combustion (lower heating value) (16.39 MJ/kg) and a high TAN number (125 mg KOH/g). In comparison, the catalytic pyrolysis biocrude had a significantly lower oxygen (24.4 wt% wet basis, 18.7 wt% dry basis) and water (9.1 wt%) content, resulting in a higher net heat of combustion (26.09 MJ/kg). The hydrothermal liquefaction biocrude contained about 22% oxygen (wet wt%) or 15.5 wt% (on a dry basis), had a water content of 8.9 wt% and a net heat of combustion at 27.40 MJ/kg. The pH of the hydrothermal liquefaction biocrude was the highest at pH 4.53.

**Table 1:** Comparison of the characteristics of the fast, catalytic and HTL biocrudes

	Units	Testing method	Fast pyrolysis biocrude	Catalytic pyrolysis biocrude	Hydrothermal liquefaction biocrude
Density @ 15 °C	kg/m^3^	ASTM D4052	1198	1163	1169.2^a^
Specific gravity 60/60F		ASTM D4052	1.197	1.164	–
Elemental analysis					
C	wt%	ASTM D5291	44.1	64.6	70.4
H	wt%	ASTM D5291	7.5	7.3	7.44
N	wt%	ASTM D5291^b^	0.132	0.16	0.12
S	ppm	ASTM D5453	84	360	1050^c^
O (dry basis)	wt%	In-house Elementar	35.6	16.5	14.5
Water content by Karl-Fisher	wt%	ASTM E203	23.5	9.1	8.91
Heat of combustion (net)	MJ/kg	ASTM D240	16.39	26.09	27.40
Total acid number (TAN)	mg KOH/g	ASTM D664	125	82.6	28.6
Kinematic viscosity at 40 °C	cSt	ASTM D445	20.67	236.4	503.4^d^
Ash content	wt%	ASTM D482	0.013	0.93	0.61
Pyrolysis solids content	wt%	ASTM D7579	0.03	1.32	1.48
pH	pH	In-house	2.66	3.00	4.53^e^
Flash point	°C	ASTM D93	50.5	< 40.0	Not detected^f^
Pour point	°C	ASTM D5460	− 36	− 6	33
Aromaticity by ^13^C NMR	%	ASTM D5461	42.9	63.9	60.9
Total carbonyl	mol/kg	ASTM E3146	4.5	3.2	Not determined

^a^Density @ 28 °C, in-house (Helpyc)

^b^ASTM D5762

^c^ASTM D4294

^d^Kinematic viscosity at 80 °C

^e^pH at 21 °C, ASTM D1293C

^f^No flash was detected before the sample began to boil

Analysis indicated that the fast pyrolysis biocrude had a very low ash (0.013%) and solids content (0.03%) compared to the other two biocrudes, likely due to filtration carried out at the BTG facility as it is a commercial facility. The higher ash content (0.93 wt%) and pyrolysis solids (1.32 wt%) of the catalytic pyrolysis biocrude was shown to be due to the zeolite catalyst used during biocrude production. As a result, PNNL lowered the solid content, via filtration through a 5 µm filter, to < 0.05% before hydrotreating. This resulted in about a 5% weight loss of bio-oil, including solids and residual bio-oil in the filter body. The hydrothermal liquefaction biocrude also had a high solids content (1.48%) and contained potassium, iron, sodium, calcium, silica, alumina, sulphur and phosphorus. Consequently, this biocrude was also filtered by PNNL before hydrotreatment. An approximate 1.1% weight loss of biocrude occurred during this process, including solids and some residual biocrude in filter cake. As it had been previously reported that the ash in biocrudes can contribute to polymerization, it is important for the stability of the biocrude that the ash be removed [13, 22, 23].

The various values reported in Table 1 for the fast and catalytic pyrolysis biocrudes were quite similar to earlier reports [24, 25]. However, the biocrude obtained via hydrothermal liquefaction (HTL) showed some properties, such as the pH and the oxygen content, that were different from previously reported values [17, 26]. It is likely that these differences were a result of the subcritical process conditions used by Aarhus compared with the HTL produced under supercritical conditions. In related work, other workers have shown that the product yield and quality of hydrothermal liquefied biocrudes is influenced by various factors such as the type of biomass and catalyst used, as well as residence time and biomass-to-solvent ratio [27]. This highlights the difficulty and complexity of defining a “typical” HTL biocrude. Other notable differences between the three biocrudes were the viscosity and aromaticity. As anticipated, the HTL biocrude had the highest viscosity (as also found in an earlier study [27]) while the aromaticity of the catalytic pyrolysis biocrude, at 63.9%, was similar to the HTL biocrude (60.9%) and was much higher than that of the fast pyrolysis biocrude (42.9%). The higher aromaticity of the HTL and catalytic pyrolysis biocrudes could potentially impact the characteristics of any upgraded fuels. For example, if low aromaticity products are required the aromatic rings need to be opened up which will require increased severity in hydroprocessing [28].

During fast and catalytic pyrolysis, liquid (biocrude), solid (char) and gaseous products are typically formed [12]. As discussed in more detail in the subsequent life cycle assessment (LCA) section, the char and gas are by-products that can be used to dry the feedstock and supply energy during production. Catalytic pyrolysis can also result in a significant aqueous fraction containing dissolved polar compounds, resulting in a loss of carbon yield in the liquid biocrude fraction. Although some researchers have suggested using aqueous phase reforming of this stream to produce hydrogen [29], this fraction has not been utilized to date. As discussed later, the relatively low yield of the catalytic pyrolysis biocrude had a significant impact on the calculated LCA values. The HTL biocrude production also resulted in a significant aqueous fraction which had a high biological oxygen demand (BOD) indicating that some form of treatment will be required before discharging the water into a sewer system. Alternatively, some researchers have suggested recycling process waters to improve biocrude yields [30–32]. Despite the limitations imposed by the variabilities that can result from changes in the configuration and conditions followed by the three different thermochemical liquefaction processes, they were shown to be fairly representative of the types of biocrudes is expected to be produced by these processes. As discussed below, each of the biocrude feedstocks proved to have both strengths and weaknesses when it came to their upgrading to finished fuels and the biojet fuel fraction in particular.

### Comparison of the two hydrotreating approaches to upgrading biocrudes

Both the CanmetEnergy-Ottawa (Canada) and PNNL, Richland (USA) laboratories have been working on upgrading of biocrudes for many years [13, 33–35]. As described in more detail in “Methods”, CanmetEnergy-Ottawa (Canmet) used a co-processing strategy, where the biocrudes were first mixed with furnace fuel oil (~ 87 wt% C, 14 wt% H, < 0.15 wt% N, and < 65 ppm S) at an 18% blend prior to hydrotreating. In contrast, PNNL used a “dedicated” hydrotreatment to process pure biocrude. Although similar reactor and operating conditions were routinely used by Canmet to upgrade the biocrudes, as described in “Methods”, higher pressures and catalyst concentrations were needed to upgrade the HTL biocrude. In contrast, PNNL used a two-step hydrotreating approach to upgrade the fast pyrolysis biocrude while a single step hydrotreatment was used for each of the catalytic pyrolysis and HTL biocrudes.

It should be noted that the upgrading mandate for the two laboratories was to use their “best” approach/method for upgrading towards ~ 0% oxygen content. Opportunities to attempt any type of optimization were very limited, thus the upgrading results essentially represent one data set. When the refined biocrudes after upgrading were assessed (Table 2), it was apparent that the oxygen content of the biocrudes after hydrotreatment and the degree of deoxygenation varied for each of the biocrudes and upgrading methods. Two of the refined biocrudes still had > 1% oxygen, indicating that more severe conditions will likely be required to completely deoxygenate these biocrudes.

**Table 2:** Composition and characteristics of biocrudes after upgrading

	Fast pyrolysis		Catalytic pyrolysis		HTL	
	Canmet	PNNL	Canmet	PNNL	Canmet	PNNL
Carbon (wt%)	84.1	87.09	85.8	88.27	85.77	88.67
Hydrogen (wt%)	13.2	12.84	13.4	10.77	13.85	11.61
Nitrogen (wt%)	b.d.	b.d.	b.d.	0.015	< 0.75	b.d.
Oxygen (wt%)	0.51	0.64	1.24	0.95	1.78	< 0.5
Sulfur (wt%)	0.13	b.d.	b.d.	b.d.	0.13	< 0.05
TAN (mg KOH/g)	0.32	b.d.	0.48	n.d.	1.35	b.d.
Density @ 15 °C (kg/m^3^)	828.6	845.6	838.7	857	838.7	899.9
Heat of combustion (net) (MJ/kg)	45.5^a^	42.366	45.21^a^	42.9	43.2^a^	42.131

*b.d.* below detection, *n.d.* not determined

^a^Gross heat of combustion (higher heating value)

When the product distribution (naphtha, jet, diesel, heavy fuel oil) in the refined biocrude after distillation was assessed (Table 3), the product distribution in the case of Canmet co-processed hydrotreatment was solely based on the biogenic fraction measured by C14 analysis. It should be noted that the product distribution of fractions was influenced by the composition of the furnace fuel oil used for blending with the biocrude prior to upgrading. As the furnace fuel oil itself contained a > 40% jet fraction this affected the resulting product distribution. Therefore, the reported fractions and distribution used for the LCA study are based on just the biogenic carbon content as this more accurately reflected the hydrotreating impact on the biocrude feedstocks.

**Table 3:** Yield of the various fractions after upgrading

Fuel fractions (%)	Fast pyrolysis		Catalytic pyrolysis		HTL	
	Canmet	PNNL	Canmet	PNNL	Canmet	PNNL
Naphtha (IBP-155 °C)	19.0	30.4	15.1	27	2.5	18.8
Jet fuel fraction (155–250 °C)	20.0	24.7	31.6	36.6	29.8	22.9
Heavy middle distillates (250–345 °C)	47.7	24.4	33.8	25.6	40.9	28.8
Heavy gas oils (+345 °C)	13.3	20.5	19.5	10.3	26.8	29.5

It was apparent that the product distribution for the different biocrudes and upgrading pathways showed very significant differences with the highest biojet fractions observed for the VTT catalytic fast pyrolysis biocrude for both upgrading methods (31.6% via co-processed hydrotreatment versus 36.6% for dedicated hydrotreatment). A significant heavy middle distillates fraction was also detected, reaching as high as 47.7% for the BTG biocrude after co-processing upgrading. The heavy fractions with a boiling point greater than 345 °C formed a substantial component in all of the refined biocrudes (~ 10–30%) although the catalytic pyrolysis (upgraded by PNNL) and fast pyrolysis (upgraded by Canmet) had lower fractions of 10.3% and 13.3%. It should be noted that increased cracking of these heavy fractions could potentially increase the biojet fraction yield.

### Preliminary evaluation of the biojet fractions after upgrading

A significant biojet fraction, from 20 to 36.6%, was obtained when each of the biocrudes was upgraded by each pathway. This was a much higher yield than the typical ~ 10% jet fraction obtained after fossil crude oil refining and indicated the potential of thermochemical liquefaction processes for production of substantial volumes of biojet fuels [36]. However, it is important that the quality of the biojet fraction is acceptable as the aviation sector uses high specification fuels to power jet turbine aircraft. This is usually classified as Jet A/A1 fuels, with these fuels meeting strict specifications defined by the jet engine and airframe OEMs documentation and approved by the regulatory authorities. Examples of fuel specifications are ASTM 1655 and Def Stan 91-91 [37], with a separate standard, ASTM D7566, created for alternative jet fuels, such as biojet fuel, to ensure that the same high standards are maintained [38]. ASTM D7566 consists of a general section with specifications listed for conventional jet fuel blended with alternative fuels such as biojet. Each alternative jet fuel has a separate specification in the Annexes to ASTM D7566 for pure/neat biojet fuel. It is worth noting that the specifications in the Annexes are adapted based on the specific chemistry of biojet technologies. Although these standards may be lower than the blended jet fuel, blending can overcome these limits such that the final blend complies fully with specifications. In fact, after a blend has been specified according to ASTM D7566, it is considered a fuel under specification ASTM D1655 and equivalent to a conventional jet fuel.

Although the biojet fuels produced via the biocrude production and upgrading pathways described here are not currently included as an annex in ASTM D7566, it is highly likely that certification will eventually take place to allow the use of biocrude-based biojet fuel in the aviation sector. Thus, we used the general specifications and analytical procedures specified in ASTM D7566 to broadly assess the suitability of these different pathways to produce biojet fuels. ASTM D7566 defines the minimum and maximum specifications of the biojet fuels after blending with conventional jet fuel in Table 1 [38]. As summarized in Table 4, when the properties of the various biojet fractions were compared with the ASTM standards it was apparent the observed variances could be overcome through further optimization of the hydrotreating and additional polishing steps, e.g. further deoxygenation; as well as through blending with fossil jet fuels. While the sulphur levels appear to meet specifications, the oxygen and nitrogen components need to be addressed. Although nitrogen specifications are limited to 2 mg/kg, further hydrotreatment would likely remove most of the nitrogen while the oxygen could be reduced to a lower level via optimization of the hydrotreater operation, also increasing the density and heat of combustion. Although excellent results were obtained for freezing points, the flash point was a little low for the upgraded HTL biocrude. However, this is likely a function of the distillation cut point and could be adjusted by removing some of the lower boiling components to raise the flash point. As the variation from specifications for the smoke point was likely due to high levels of aromatics which caused a higher carbon to hydrogen ratio, additional hydrogenation and hydrocracking will probably be needed to further reduce the aromatic content. It is worth noting that most of the biojet fuels described in the ASTM D7566 Annexes have a very low aromatic content. Through blending of the biojet fuel with a fossil jet fuel, an aromatic content within specifications can be achieved. In contrast, the biojet fractions in this study all had a high aromatic content (between 14 and 30%), even after hydrotreatment upgrading. Thus, to meet the blend specification of 25% aromatics, consideration must be given to substantially reducing aromatics in the biojet fractions produced and reported here. Current approved biojet fuels as described in the Annexes, including Fischer–Tropsch (FT), hydrotreated esters and fatty acids (HEFA), synthetic isoparaffins (SIP) and alcohol to jet (ATJ) have a limit of 0.5% aromatics within the standard. It is worth noting that thermochemical liquefaction-based fuels are likely to generate high levels of aromatics based on the lignin content of the lignocellulosic feedstocks. It was apparent (Table 1) that the biocrudes produced through catalytic pyrolysis and hydrothermal liquefaction have a much higher aromatic content (~ 60%) than fast pyrolysis biocrude and would likely have to be reduced through more aggressive hydrocracking to break down the aromatic rings.

**Table 4:** How the characteristics of the various biojet fractions compare with ASTM D7566 specifications (listed in Table 1)

	Fast pyrolysis		Catalytic pyrolysis		HTL	
	Canmet	PNNL	Canmet	PNNL	Canmet	PNNL
Composition						
Acidity, total mg KOH/g Max 0.10	0.064	*0.11*	*0.101*	0.012	0.100	0.014
Aromatics, volume percent Max 25	17.7	18.6	19.3	*30.4*	14.1	20.9
Sulfur, mercaptan, mass percent Max 0.003	0.0019	< 0.0003	0.0003	0.0021	Nd	< 0.0003
Sulfur, total mass percent Max 0.30		< 0.25		< 0.25	0.0518	< 0.25
Volatility						
Flash point, °C Min 38	61	43	58.5	*34.5*	59.0	*34.5*
Density at 15 °C, kg/m^3^ 775 to 840	818.9	*843.4*	827.8	*852.6*	829.0	*853*
Fluidity						
Freezing point, °C Max − 47 Jet A-1*I*	− 57.6	− *46.7*	− 58.3	< − 80	− *45*	− 84
Viscosity − 20 °C, mm^2^/s, Max 8.0	5.164	5.176	5.306	3.499	6.6	4.431
Combustion						
Net heat of combustion, MJ/kg Min 42.8	42.92	*42.245*	*42.32*	*42.477*	42.85	*42.55*
One of the following requirements shall be met: (1) Smoke point, mm, or Min 25.0 (2) Smoke point, mm, Min 18.0 and naphthalenes, volume, percent Max 3.0	21	18.3	20	*14.3*	19.7	*17*
	0.51	2.17	1.51	0.36	1.07	0.44
Corrosion						
Copper strip, 2 h at 100 °C Max No. 1 (3 is off spec)	1b	1a	*3a*	*3b*	1a	*3a*
Contaminants						
Existent gum, mg/100 ml Max 7	*15*	*28*	*65*	< 1	*41*	3
Nitrogen, mg/kg Max 2		*270*		*9.7*	< 0.15	*81*
Water, mg/kg Max 75	*138*	*440*	*159*	66	*79*	74
Sulfur, mg/kg Max 15	*480*	11	*423*	*39.3*	0.0518	
Oxygen wt%	0.3	1.08	1.18	< 0.01	2.42	0.13

Off-specs are italicized

### Life cycle assessment

As discussed earlier, the aviation sector wants to reduce its carbon footprint and this is a major reason why biojet fuel development is being pursued so aggressively. However, it is important that the carbon intensity of the biojet fuel is assessed and documented as the carbon reductions can then be used to meet targets or calculate offsets under the ICAO CORSIA scheme [39]. As described in more detail in “Methods”, the GHGenius LCA model was used to calculate the carbon intensity (CI) for both the production and upgrading of each biocrude based on the Canmet and PNNL methods. The various inputs that went into the model for each biocrude and upgrading approach are summarized in Additional file 1.

It was apparent that the production of the fast pyrolysis biocrude results in the lowest emissions (Table 5), primarily as a result of high yields and minimal fossil energy inputs. The other two biocrudes showed similar, but much higher emissions, although the emission profiles were quite different. For example, production of the catalytic pyrolysis biocrude showed high feedstock emissions due to the low overall yield of biocrude per tonne of feedstock, while the HTL biocrude had low feedstock emissions but high process emissions due to the higher fossil energy input into the system to achieve the appropriate production conditions (pressure and temperature).

**Table 5:** GHG emission comparison of biocrude and refined biocrude after upgrading (g CO_2_eq/GJ HHV)

	Fossil jet fuel	Fast pyrolysis biocrude		Catalytic pyrolysis biocrude		HTL biocrude	
	Crude oil	Canmet	PNNL	Canmet	PNNL	Canmet	PNNL
Fuel dispensing	91	95	95	94	94	97	96
Fuel distribution and storage	642	626	626	636	636	615	652
Fuel production	6383	93,640	51,617	85,324	28,467	73,421	30,463
Feedstock transmission	78	3186	3892	5196	5446	2397	2454
Feedstock recovery	5647	3789	4629	7484	7843	3156	3230
Feedstock upgrading	4720	2461	3006	4505	4722	11,686	11,961
Land-use changes, cultivation	210	12	14	23	24	10	10
Fertilizer manufacture	0	0	0	0	0	0	0
Gas leaks and flares	2280	0	0	0	0	0	0
CO_2_, H_2_S removed from NG	0	0	0	0	0	0	0
Emissions displaced–co-products	− 138	− 19,425	− 42,038	− 14,093	− 10,846	− 9171	− 7603
Fuel production	19,913	84,384	21,841	89,170	36,386	82,211	41,263
Fuel use	67,637	626	626	626	626	626	626
Total (g CO_2_ eq/GJ)	87,550	85,010	22,467	89,796	37,027	82,837	42,889
% change		− 2.9	− 74.3	2.6	− 57.7	− 5.4	− 51.0

The potential emission reductions calculated in this study for the Canmet co-processing pathway amounted to − 2.9% for the upgraded fast pyrolysis biocrude, 2.6% for the upgraded catalytic pyrolysis biocrude and − 5.4% for the upgraded HTL biocrude which were unexpectedly low. However, this was not a true reflection of the co-processing strategy used, but rather the impact of the additives Canmet used to overcome miscibility and viscosity issues relating to the biocrudes. These miscibility issues were overcome by addition of surfactant and methanol to the mixture of biocrude and furnace fuel carrier oil to create a more homogenous micro-emulsion. Similarly, the high viscosity of the catalytic pyrolysis biocrude and the HTL biocrude was further overcome with the addition of DEGMME (diethylene glycol monomethyl ether). When the addition of these compounds was included as part of the full lifecycle assessment, the resulting emissions from upgrading were very high and very limited emission reduction could be achieved. It was apparent that the chemicals added to the Canmet system had a significant impact on the GHG emissions of the refined bio-oil as, in the current configuration, they were not recovered and recycled. When the dosage rates used were compared to biocrude volumes produced, almost 0.75 kg of chemicals was added for every kg of refined biocrude produced.

The miscibility challenges of fast pyrolysis biocrudes are well documented and multiple studies have looked at various additives as one way to overcome this problem [35, 40, 41]. However, it appears that the impact of additives on the life cycle assessment of drop-in biofuels has not previously been considered. The significant impact this may have on the potential emissions reductions of a fuel should be an important consideration for future studies, particularly those looking at co-processing strategies.

In contrast to the Canmet approach, all of the biocrudes upgraded by PNNL using dedicated hydrotreating showed significant GHG emission reductions from 51% for the HTL biocrude, 57.7% for the catalytic pyrolysis biocrude, to a 74.3% reduction for the fast pyrolysis biocrude. The much higher emission reductions calculated for the fast pyrolysis biocrude and PNNL upgrading pathway are due to the 5–6 times higher production of gaseous coproduct as compared with the other biocrudes. As it was assumed that this gas is available to replace natural gas for process energy, a larger emission credit was available for this pathway that more than offset the additional hydrogen required for upgrading. As a result, this pathway achieved the greatest overall emission reductions.

The PNNL upgrading of the other biocrudes also resulted in significant (more than 50%) emission reductions, although the contribution of the various steps differed (Table 5). It should be noted that, as the catalytic pyrolysis and HTL biocrude production processes are at a lower level of technology readiness than fast pyrolysis biocrude production, further optimization of these processes will most likely result in improved LCA values.

Related LCA work has shown that the various drop-in biofuel processes that have been studied do not have a “fixed” emission reduction potential, with significant variations resulting from factors such as the type of feedstock used, geographical location, supply chain, source of electricity, etc. Other influences on the LCA include process design and choices, including how the co-products are considered (e.g. used for generation of process energy or otherwise) and source of hydrogen (e.g. from natural gas/electrolysis or other methods) [4, 42]. For example, other workers have shown that the source of hydrogen has a significant impact on emission reductions with hydrogen generated from bio-char resulting in a 45% reduction in the GHG emissions [3]. In the work described here, as the biocrude production and upgrading pathways were not optimized it is highly likely that improved LCA reductions can be achieved in future.

It is also recognized that the source and characteristics of the biomass feedstock will have an impact on the LCA [43–45]. For example, when forest residues are used (as modelled in this study) additional energy will likely be required for comminution and transport, whereas mill residues (such as sawdust) have no transport cost from the forest and, typically, no further size reduction is required [44]. In addition, drying of mill residues will not be required while forest residues will likely require a reduction in moisture content for the fast and catalytic pyrolysis processes. However, where sufficient biochar is produced during the pyrolysis, it could be used to reduce the additional energy inputs for drying. Thus, the LCA of a particular pathway will not remain static and further modifications will likely have a significant impact on the LCA values determined.

**Fig. 1:**
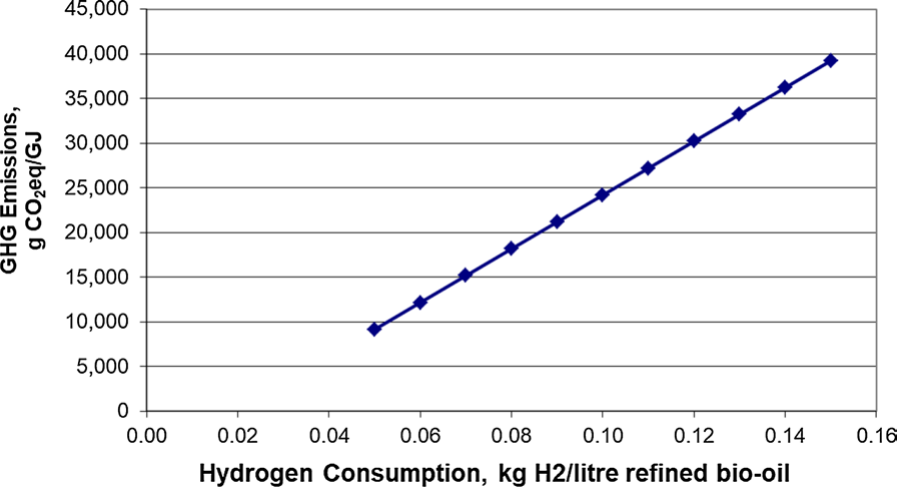
Sensitivity to hydrogen consumption

Based on a sensitivity analysis of the HTL process, the largest contributor to the overall GHG emissions at the upgrading stage was from hydrogen consumption. For the LCA modelling studies, varying the hydrogen base value requirements from 50 to 150% (equivalent to 0.10 kg H_2_/l refined biofuel) showed the impact of hydrogen consumption to be significant (Fig. 1).

The type and source of biomass feedstock also has a significant impact on LCA results [45, 46]. For the pathways assessed in this study the softwood feedstocks were assumed to be British Columbia forest residues which are currently burned in the forest to meet BC forest management regulations [47]. However, it has been shown that, due to the poor combustion characteristics of the open pile burning, there are significant GHG emissions associated with this practice. If the avoided emissions from current practices are included in the LCA assessment, the potential emission reductions would become much greater. Thus, processes such as catalytic pyrolysis that tend to use more feedstock would have even higher avoided emissions as a result of their lower biocrude production yields.

Previous work [4] that compared biojet fuel production from biocrudes produced by pyrolysis or HTL of forest residue feedstock showed potential GHG emissions reductions of 66.5–69.5% for the HTL-derived biojet fuel and 46.4–65.5% for the pyrolysis-derived biojet fuel, as compared to fossil jet fuel. In related LCA work [19] a 53.5% and 60.5% reduction for pyrolysis and HTL derived biojet fuels, respectively, was reported while other work claimed a 68–76% reduction when using pyrolysis biocrudes derived from corn stover feedstocks [6]. Other LCA work that looked at the pyrolysis of logging residues showed a reduction of 59–62% when using the GREET or SimaPro LCA models, with greater reductions obtained using corn stover feedstock [48].

In the work reported here fast pyrolysis showed the greatest emission reductions potential (74.3%) while HTL showed a potential reduction of 51%. Several factors can greatly influence these values from the geographical location of the plant to the carbon intensity of the electricity that is used. However, all of the studies to date that have looked at producing drop-in biofuels from upgraded biocrudes have all shown the potential for such routes to achieve substantial benefits in terms of lowering the carbon intensity of biofuels, including the biojet fuel fraction.

## General discussion

As the refined biocrudes contained various levels of oxygen, we were not able to compare the effectiveness of upgrading with regard to hydrogen consumption. Although hydrogen consumption has been shown to have a significant impact on the life cycle assessment (Fig. 1) [3, 4, 49], the fast pyrolysis pathway showed the highest level of emission reductions potential despite also requiring the most hydrogen for upgrading (Table 5). As one of the biggest challenges reported for using fast pyrolysis biocrudes is its high oxygen content, several researchers have advocated for the production and use of catalytic pyrolysis-derived biocrudes due to their potential to contain a substantially lower oxygen content and thus requiring much less hydrogen for upgrading [46]. However, other researchers have argued that this is an oversimplification and that it is not merely the presence of oxygen (or its concentration) but the “way in which the oxygen is present in the bio-oil” that is important [14]. For example, oxygen in the form of alcohols and ethers would be far more favourable than having oxygen present in the form of carboxylic acids, aldehydes or ketones since various combinations of acids/alcohols and alcohols/aldehydes can react with each other to polymerize [14].

In the work reported here, although the catalytic pyrolysis biocrude had a significantly lower oxygen content (16.5% dry basis) than the fast pyrolysis biocrude (36.5% dry basis), this came at the expense of yield, with a substantial amount of the carbon lost to the aqueous phase (Table 6). In addition, the lower oxygen in both the catalytic pyrolysis and HTL biocrudes (14.5% dry basis) did not appear to improve its miscibility in the co-hydrotreating strategy plus their greater viscosity resulted in additional processing challenges.

**Table 6:** Summary of upgrading three biocrudes via two approaches

	Fast pyrolysis biocrude		Catalytic pyrolysis biocrude		HTL biocrude	
	Canmet	PNNL	Canmet	PNNL	Canmet	PNNL
Biocrude—oxygen content (%) (dry)	35.6		16.5		14.5	
Biocrude—kg wood/l biocrude	1.88		6.55		3.05	
Biocrude—kg wood/MJ biocrude	0.087		0.203		0.085	
Total yield of biocrude and upgrading (wt%)	23	19	12	11	26	27
Potential emission reduction refined biocrude (%)	− 2.9	− 74.3	2.6	− 57.7	− 5.4	− 51.0
Yield—kg wood/l refined biocrude	4.14	5.08	8.19	8.58	3.27	3.63
Hydrogen consumption kg/l refined biocrude	0.180	0.163	0.115	0.091	0.07	0.101
Remaining oxygen in refined biocrude wt%	0.3	1.08	1.18	< 0.01	2.42	0.13
Higher heating value (HHV) refined biocrude (MJ/l)	37.72	37.46	38.39	35.57	36.24	39.40
Jet fraction (%)	20.0	24.7	32.8	36.6	29.8	22.9

Overall, the catalytic pyrolysis biocrude pathway resulted in the lowest biocrude yields, but the highest final yields after upgrading (Table 6). This pathway also resulted in the largest jet fractions and a reasonable emission reduction potential when using the PNNL upgrading pathway (− 57.7%). Although the fast pyrolysis biocrude started with a very high oxygen content, it delivered good overall yields and the best emission reduction potential when upgraded by PNNL (− 74.3%) as well as resulting in good jet yields (Table 6). The hydrothermal liquefaction biocrude delivered the highest yields and also showed substantial emission reductions (− 51% for the PNNL pathway). However, a likely challenge for HTL commercialization will be the higher pressures required during biocrude production and the associated engineering challenges.

## Conclusions

Three biocrudes produced via fast pyrolysis, catalytic pyrolysis and hydrothermal liquefaction of softwoods were subsequently upgraded to drop-in fuels and fractionated into multiple fuel products including biojet fuel. Upgrading was carried out via two distinct methodologies that included “dedicated” hydrotreating (PNNL) and co-processed hydrotreating (Canmet). All of the pathways produced a significant biojet fuel fraction (20–36%) which was significantly greater than the typical 10% jet fraction resulting from refining of fossil crude oil.

When the various biojet fractions were compared against some of the main specifications listed under ASTM7655 (for blended fuels), a significant level of compliance was apparent. Although some specifications were outside the ASTM7655 specification it is likely that further upgrading and optimization, e.g. removal of residual oxygen, will improve the quality of the biojet and comply with specifications. The highest potential emission reductions (74%) were achieved using the fast pyrolysis biocrude upgraded by PNNL via their two-stage dedicated hydrotreating approach. However, both the catalytic pyrolysis and HTL biocrudes, when upgraded by PNNL, also showed significant potential emissions reductions of 57% and 51%, respectively. Dedicated hydrotreatment, as demonstrated by PNNL, was more successful with respect to emission reductions compared with the co-processing hydrotreatment approach. This was primarily due to the addition of chemicals required to ensure miscibility and reduce the viscosity of the liquid bio-intermediates. It is highly likely that a reduction in the addition of these chemicals, through optimization or substitution with renewable alternatives, will significantly improve the life cycle performance of the co-processing hydrotreatment pathway. The production of biojet fuels based on direct thermochemical liquefaction-derived biocrudes upgraded by hydrotreating appears to have considerable potential.

## Methods

### Biocrudes

The three biocrudes were purchased as representative examples of different biocrude production technologies. Two 50-l volumes of each biocrude were obtained to allow sufficient volumes of upgraded product (50 l to each upgrading laboratory) to be produced to enable further analysis. All of the biocrudes were produced from softwood feedstocks, sufficiently similar to allow comparison. The fast pyrolysis biocrude was obtained from BTG, Hengelo, the Netherlands (fast pyrolysis (FP) biocrude), the catalytic pyrolysis biocrude from the VTT Technical Research Centre of Finland, Espoo, Finland (catalytic fast pyrolysis (CFP) biocrude) and the hydrothermal liquefaction biocrude from Aarhus University, Denmark (hydrothermal liquefaction (HTL) biocrude), with the process operated under subcritical conditions. The FP biocrude was produced as described on the BTG website [24]. The CFP biocrude was produced as described in Paasikallio et al. [25]. The HTL biocrude was produced according to the method described in Anastasakis et al. [32].

These three biocrudes were the primary products of direct thermochemical liquefaction, each comprising fractions of hydrocarbon liquid intermediates that can be used for upgrading into drop-in hydrocarbon fuels. As a general principle, thermochemical technologies such as pyrolysis and gasification produce three main fractions including gas, char and liquid. The higher temperatures (> 800 °C) employed during gasification favour the production of gas, while the lower temperatures (~ 500 °C) employed during pyrolysis favour the production of a hydrocarbon liquid fraction [11]. In addition, processes such as catalytic pyrolysis also produce an aqueous phase as a result of water formation during hydrodeoxygenation. Hydrothermal liquefaction also produces an aqueous phase containing higher levels of carbon than the catalytic pyrolysis. For production of drop-in biofuels, the hydrocarbon liquid (bio-oil or biocrude) is the main fraction of interest as it can be further upgraded into finished fuels. Biocrudes were shipped to the Canmet or PNNL labs with analysis of the biocrudes carried out prior to upgrading at the respective laboratories.

### Upgrading through hydrotreatment

CanmetENERGY-Ottawa’s (Canmet) upgrading approach involved co-hydroprocessing of biocrude in a one-stage reactor with furnace fuel oil (reaction medium) using a highly dispersed unsupported molybdenum sulfide (MoS2) catalyst that is generated in situ from an emulsified precursor [35]. The biocrude and reaction medium mixtures were prepared as microemulsions prior to injection into the hydrotreatment reactor. The preparation of the biocrude microemulsion was adapted from the method used by Ikura et al. [35] for making stable microemulsions with 5–30 wt% bio-oil in diesel fuel. For the VTT CFP and Aarhus U. HTL biocrude microemulsions, a diluent (diethylene glycol monomethyl ether, DEGMME) was added to reduce the viscosity of the biocrude prior to preparing the microemulsions and higher levels of surfactant were used for these biocrude blends (average 4 wt% vs. 2.7 wt% for processing FP biocrude). As the HTL biocrude was solid at room temperature, it was heated to 70 °C before mixing with DEGMME, furnace fuel oil and the surfactant solution. The catalyst precursor solution and the sulphiding agent (tertiary-butyl polysulfide—TBPS) were subsequently mixed with each biocrude microemulsion prior to feeding into the reactor system.

The biocarbon content of the liquid products (combined oil product and four fractions obtained by spinning band distillation) was determined using ^14^C analysis (radiocarbon method) on a 3MV tandem accelerator mass spectrometer (AMS) built by High Voltage Engineering Europa B.V. (HVE). The biocarbon contents were calculated relative to the carbon ^14^C of the corresponding biocrude [50].

Pacific Northwest National Laboratories used a dedicated hydrotreatment approach to upgrade the biocrudes. All three biocrudes were hydrotreated in the same reactor system, but in separate tests. The reactor system was built around a continuous, down-flow packed-bed reactor loaded with a commercial Ni–Mo sulfide-based hydrotreating catalyst. General information about the reactor and method is described in Olarte et al. [51]. The FP biocrude was hydrotreated in a two-step process with an initial low-temperature hydrogenation step used to stabilize the biocrude prior to the high-temperature hydrodeoxygenation/hydrocracking step [13]. CFP and HTL biocrudes were hydrotreated in one step without initial stabilization.

### Life cycle assessment

To provide an effective LCA comparison between the various technology pathways, a common feedstock supply chain was assumed, based on forest residues sourced in British Columbia, Canada. The modelled supply chain assumed that the forest residues were comminuted in the forest and transported by truck to the biocrude facility (assumed to be a 200 bbl per day biocrude production facility). The biocrude was then transported by rail to Vancouver where upgrading in a small-scale hydrotreater, co-located with an existing petroleum refinery, occurred. Downstream distribution of fuel products was assumed to use the existing refinery distribution channels. Based on the annual biomass demand for the biocrude production facility, a feedstock cost of $80/dry tonne within a 100-km supply radius was assumed.

The GHGenius LCA model 5.0c was used to carry out the LCA [52] with the year set to 2018 and the region to British Columbia. All other user inputs were set to their default values unless otherwise specified. Petroleum-based jet fuel was used as a reference.

The assessment considered only the GHG emissions associated with the production and use of the refined biocrudes. The GHG emissions were calculated using the 100-year global warming potentials from the 2007 IPCC fourth assessment report [53], as these are the values currently being used for government reporting: carbon dioxide = 1; methane = 25; nitrous oxide = 298. To ensure consistency with IPCC methodology, the GHG emissions of carbon monoxide and unburned hydrocarbons were calculated based on the assumption that these short-lived gases are oxidized to carbon dioxide. The emissions from each of the processes were allocated to the components based on their energy content. The system boundary starts with the collection of the forest residue and ends with the use of the refined biocrude in an aircraft.

## Supplementary information

**Additional file 1.** Additional information regarding assumptions and input for life cycle assessment.

## Data Availability

The detailed data used for this study will be soon available in a full report posted on the IEA Task 39 website (http://task39.ieabioenergy.com/). The input data for LCA study is available in the additional document and the GHGenius model is available online [52].

## Abbreviations

ASTM: American Society for Testing and Materials
SAF: sustainable aviation fuels
FOG: fats, oils and grease
PNNL: Pacific Northwest National Laboratories
TAN: total acid number
HTL: hydrothermal liquefaction
LCA: life cycle assessment
BOD: biological oxygen demand
FT: Fischer–Tropsch
HEFA: hydrotreated esters and fatty acids
SIP: synthetic isoparaffins
ATJ: alcohol to jet
ICAO: International Civil Aviation Organization
CORSIA: Carbon Offsetting and Reduction Scheme for International Aviation
CI: carbon intensity
TBPS: tertiary-butyl polysulfide
DEGMME: diethylene glycol monomethyl ether
FP: fast pyrolysis
CFP: catalytic fast pyrolysis
