# Supplementary material for: Potential yields and emission reductions of biojet fuels produced via hydrotreatment of biocrudes produced through direct thermochemical liquefaction
Source: Biotechnol Biofuels. 2019 Dec 5;12:281. doi: 10.1186/s13068-019-1625-2 (PMC6894131; doi:10.1186/s13068-019-1625-2)
Supplement: Supplementary file 1 — Additional file 1. Additional information regarding assumptions and input for life cycle assessment. [file 13068_2019_1625_MOESM1_ESM.docx]

Additional file 1 – Additional information regarding assumptions and input for life cycle assessment

Table S1: Input for biocrude production

|  | BTG Biocrude | VTT Biocrude | HTL Biocrude |
| --- | --- | --- | --- |
| Density, kg/litre | 1.197 | 1.168 | 1.120 |
| Oxygen, % | 47.5 | 16.5 | 14.5 |
| MJ/litre, (HHV) | 21.5 | 32.3 | 35.9 |
| kg wood/litre oil | 1.88 | 6.55 | 3.05 |
| kg wood/MJ oil | 0.087 | 0.203 | 0.085 |
| MJ gas/MJ oil | 0.62 | 0.38 | 0 |
| kWh/litre oil | 0.10 | 0.20 | 0.20 |
| NG, MJ/litre oil | 0.5 | 0 | 7.7 |
| Nitrogen, kg/litre oil | 0.035 | 0 | 0 |
| Other, per litre oil | 0 | 0.012 kg ZSM-5 | 0.0305 kg KOH |
|  |  |  | 0.0076 kg CMC |

Table S2: LCA of biocrude production

|  | Fast pyrolysis Biocrude | Catalytic pyrolysis Biocrude | HTL Biocrude |
| --- | --- | --- | --- |
|  | Forest Residue | Forest Residue | Forest Residue |
|  | g CO_2_eq/GJ | g CO_2_eq/GJ | g CO_2_eq/GJ |
| Fuel dispensing | 0 | 0 | 0 |
| Fuel distribution and storage | 0 | 0 | 0 |
| Fuel production | 2,320 | 5,008 | 12,910 |
| Feedstock transmission | 2,277 | 5,303 | 2,222 |
| Feedstock recovery | 3,572 | 8,319 | 3,486 |
| Feedstock upgrading | 0 | 0 | 0 |
| Land-use changes, cultivation | 11 | 26 | 11 |
| Fertilizer manufacture | 0 | 0 | 0 |
| Gas leaks and flares | 0 | 0 | 0 |
| CO_2_, H_2_S removed from NG | 0 | 0 | 0 |
| Emissions displaced - co-products | 0 | 0 | 0 |
| **Fuel Production** | **8,180** | **18,656** | **18,629** |

Table S3: input for upgrading fast pyrolysis biocrude

|  | PNNL | CanMet |
| --- | --- | --- |
| Feed, kg biocrude/litre RBO | 2.70 | 2.21 |
| Surfactant, kg/litre refined biocrude | 0 | 0.34 |
| Methanol, kg/litre refined biocrude | 0 | 0.14 |
| TBPS, kg/litre refined biocrude | 0 | 0.13 |
| Hydrogen, kilogram/litre HBO | 0.163 | 0.180 |
| Gaseous co-product, MJ/litre RBO | 26.77 | 12.37 |

Table S4: input for upgrading catalytic pyrolysis biocrude

|  | PNNL | CanMet |
| --- | --- | --- |
| Feed, kg biocrude/litre RBO | 1.31 | 1.07 |
| Surfactant, kg/litre refined biocrude | 0 | 0.28 |
| Methanol, kg/litre refined biocrude | 0 | 0.11 |
| TBPS, kg/litre refined biocrude | 0 | 0.08 |
| DEGMME, kg/litre refined biocrude | 0 | 0.31 |
| Hydrogen, kilogram/litre HBO | 0.092 | 0.115 |
| Gaseous co-product, MJ/litre RBO | 7.08 | 9.2 |

Table S5: input for upgrading HTL biocrude

|  | PNNL | CanMet |
| --- | --- | --- |
| Feed, kg biocrude/litre RBO | 1.19 | 1.07 |
| Surfactant, kg/litre refined biocrude | 0 | 0.25 |
| Methanol, kg/litre refined biocrude | 0 | 0.10 |
| TBPS, kg/litre refined biocrude | 0 | 0.10 |
| DEGMME, kg/litre refined biocrude | 0 | 0.27 |
| Hydrogen, kilogram/litre HBO | 0.101 | 0.070 |
| Gaseous co-product, MJ/litre RBO | 5.09 | 9.2 |
